# The evaluation of a scoring system in airway management after oral cancer surgery

**DOI:** 10.1186/s40902-015-0021-5

**Published:** 2015-07-29

**Authors:** Ho-Jin Lee, Jin-Wook Kim, So-Young Choi, Chin-Soo Kim, Tae-Geon Kwon, Jun-Youg Paeng

**Affiliations:** 1 Department of Oral and Maxillofacial Surgery, Sahmyook Adventist Dental Hospital, Seoul, Republic of Korea; 2 Department of Oral and Maxillofacial Surgery, School of Dentistry, Kyungpook National University, 2175 Dalgubeoldae-ro, Daegu, 700-705 South Korea

**Keywords:** Tracheostomy, Oral cancer, Scoring system, Nasotracheal intubation

## Abstract

**Background:**

The purpose of this retrospective study was to investigate the usefulness of tracheostomy scoring system in the decision of postoperative airway management in oral cancer patients.

**Materials and methods:**

A total of 104 patients were reviewed in this retrospective study, who underwent radical resection with or without neck dissection and free flap reconstruction due to oral cancer. The patients were classified into three groups according to the timing of the extubation; extubated groups (n = 51), overnight intubation group (n = 45), and tracheostomy group (n = 8). Cameron’s score was used to evaluate the relation between the state of the patient’s airway and the type of the operation.

**Results:**

Tracheostomy was performed in eight patients (8/104, 7.7 %). A total of 22 patients (21.2 %) had more than 5 points of which 17 patients (77.3 %) did not have a tracheostomy and any postoperative emergency airway problems. The tracheostomy scores were significantly different among the three groups. Hospital stay showed a significant correlation with the tracheostomy score.

**Conclusions:**

The scoring system did not quite agree with the airway management of the authors’ clinic; however, it can be one of the clinical factors predicting the degree of the postoperative airway obstruction and surgical aggressiveness for recovery. The further studies are needed for clinically more reliable scoring systems.

## Background

Airway management is the most important part in postoperative care after maxillofacial cancer surgery. Pulmonary complications are reported as the most common perioperative complications in microvascular head and neck reconstruction [1, 2]. Elective or emergency tracheostomy is commonly used in some clinics to secure the airway after aggressive resection of the oral cancer and simultaneous reconstruction surgery with free flaps for the defect. However, predicting the postoperative airway state and making a decision in an individual case are still difficult in clinical situations.

The morbidity after tracheostomy cannot be ignored. The tracheostomy related complication rates are reported as 4.1−45 % [3, 4]. Bleeding, obstruction of the tracheostomy tube, pneumonia, excessive scarring, and tracheal stenosis are common and these complications can result in an increased length of the patient’s recovery and hospitalization [5].

Maintaining intubation for 24–48 h postoperatively has been adopted for less extensive head and cancer surgeries to avoid a tracheostomy. If there is a possibility of having to maintain the endotracheal tube for more than 2 days, elective tracheostomy is recommended. However, it is difficult to decide which management is best for specific situations. The experience of the operator is still the most important factor in making the decision whether to perform a tracheostomy. Some objective scoring systems have been developed and tried in oral cancer surgery. Cameron et al. [6] developed a scoring system to help identify patients requiring an elective tracheostomy based on the tumor location and the types of surgery including mandibulectomy, neck dissection, and reconstruction. The authors recommend that elective tracheostomy should be considered in patients with a score higher than 5.

The purpose of this study was to analyze, retrospectively, post-operative airway management including elective tracheostomy in oral cancer patients according to Cameron’s scoring system.

## Methods

### Patients

A total of 104 patients who underwent radical resection with or without neck dissection and free flap reconstruction due to oral cancer from 2008 to 2012 at the Department of Oral and Maxillofacial Surgery, Kyungpook National University Hospital were reviewed in this study. All patients had their lesions in the oral cavity. The patients with cancer on the parotid gland, upper and lower lip, and mouth corner, which are relatively easy cases for postoperative airway management, and simple excision and primary closure cases (T1) were excluded.

The patients were classified into three groups. The patients in the extubated group were returned to maxillofacial ward without a nasotracheal tube, which was removed in the recovery room by an anesthesiologist. The overnight intubation group consisted of patients who had maintained nasotracheal intubation for one or two postoperative days. The nasotracheal tube was usually removed by the operator on the first or second postoperative day. The airway was checked with clinical examination with or without neck CT. The endotracheal tube was removed when the patient can breathe with obstruction the E-tube after deballooning. Elective tracheostomy, if necessary, was performed at the end of the operation after finishing the skin suture. The decision for postoperative airway management was made based on the operator’s experience. Usually large tumors (T4), the mouth floor or posterior lesions on the tongue and bilateral neck dissection were considered for an elective tracheostomy.

### Tracheostomy Score

A tracheostomy score, which was adopted from the scoring system recommended by Cameron (2009), was used to evaluate the state of the patient’s airway based on the type of operation (Table 1). The hospital stay of the patients was also reviewed.

**Table 1:** The tracheostomy scoring system (by Cameron, 2009) [6]

Scoring factor			Score
Tumor site	Cutaneous		0
	Mouth	Buccal mucosa	0
		Maxilla	0
		Mandibular alveolus	1
		Anterior tongue	1
		Floor of mouth	2
	Oropharynx	Soft palate	3
		Anterior pillar	3
		Tonsillar pillar	4
		Posterior tongue	4
		Hypopharynx	4
Mandibulectomy		No	0
		Yes	1
Bilateral neck dissection		No	0
		Yes	3
Reconstruction		None	0
		RFFF	2
		Other	3

*RFFF* radial forearm free flap

### Statistical analysis

The sample distribution was not normal in some groups, which was determined by the Shapiro-Wilk normality test. The Kruskal-Wallis test with multiple-comparison post test and the Mann–Whitney *U*-test were used to compare tracheostomy and hospital stay between the groups. Fisher’s exact test was used to analyze the categorical dichotomized variables and relationships. All tests were performed with the *R* (R Core Team, 2013) software package on a personal computer, and *p* < 0.05 was accepted as the level of statistical significance. This study was approved by the institutional review board of Kyungpook National University Hospital (No. 2013-12-009).

## Results

One hundred four patients were included in this review, 67 male and 37 female patients with a mean age of 60.7 ± 13.8 years (age range, 16–90 years), and their demographic details are presented in Table 2. Seventy-three patients underwent neck dissection, and 58 patients, which included 27 forearm free flaps, had microvascular reconstruction surgery. There were no statistically significant differences among the three groups in age, gender, and ASA grades (Fisher’s exact test, p > 0.05).

**Table 2:** Information related to the airway management for each group

Type of airway management	No. of patients	No. of patients more than 5 points	Duration (days, range)	Tracheostomy score (mean ± SD)	Hospital Stay (days, mean ± SD, range)
Immediate Extubation	51 (49.0 %)	6 (11.8 %)^a^	-	2.1 ± 2.0	16.6 ± 9.2 (4–46)
Overnight intubation	45 (43.3 %)	11 (24.4 %)	1.24 ± 0.67 (1–4)	2.2 ± 2.2	24.5 ± 10.1 (9–56)
Tracheostomy	8 (7.7 %)	5 (62.5 %)^a^	12 ± 8.2 (6–30)	5.4 ± 2.1	31.4 ± 16.9 (11–57)
Total	104(100 %)	22(21.2 %)			

^a^Significant difference between the Extubated group and Tracheostomy group (*P* < 0.05, multiple comparison after Fisher’s exact test with Bonferroni’s correction)

Tracheostomy was performed in eight patients (8/104, 7.7 %). Seven patients had an elective tracheostomy at the end of the operation, and one patient had an emergency tracheostomy during the postoperative period due to an airway obstruction. That patient had a pulmonary complication (pneumonia) after the tracheostomy. However, the score for the patient was 3. A summary of the patients who had a tracheostomy is presented in Table 3.

**Table 3:** Summary of the tracheostomy patients

Patients	Age	ASA class	Primary pathology	Main operation	Neck dissection	Reconstruction	Tracheostomy Score^a^
1	M/53	2	SCC alveolus (cT4N2bM0)	Segmental Resection	Lt. RND	FFF	5
2	F/69	2	SCC on tongue (cT4N1M0)	Subtotal Glossectomy	Rt. mRND	RFFF	6
3	M/49	2	SCC on mouth floor (cT2N2M0)	Segmental Resection	Rt. SOND	FFF	6
4	F/68	1	SCC on tongue (pT4N1M0)	Subtotal Glossectomy	Rt. SOND	RFFF	6
5	M/49	2	SCC on Ant. mouth floor area	Ant. Mn. resection	Both mRND	FFF	9
6	M/62	2	Osteosarcoma on Lt. Facial area (pT4N0M0)	Surgical Excision	-	-	4
7	M/48	1	SCC on Rt. Mx. area	Total maxillectomy	Both SOND	-	3
8	M/46	2	SCC alveolus	Marginal Resection	Rt. SOND	RFFF	3

*ASA* American society of anesthesiologist, *SCC* squamous cell carcinoma, *RFFF* radial forearm free flap, *FFF* fibular free flap, *Mn* mandible, *Mx* maxilla, *mRND* modified radical neck dissection

^a^Score from Cameron (2009) [6]

**Fig. 1:**
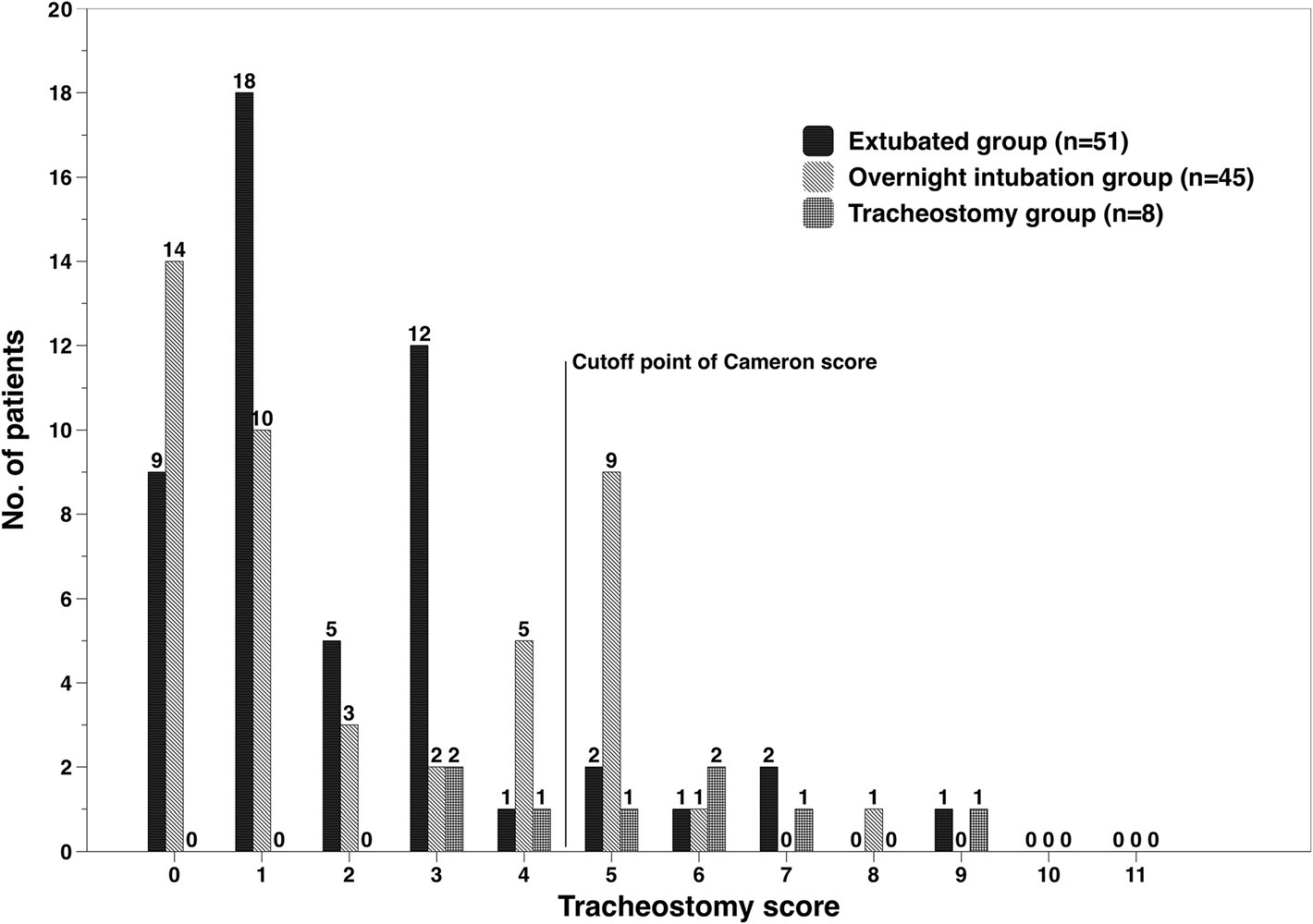
The number of patients in the airway management groups according to the tracheostomy score

**Fig. 2:**
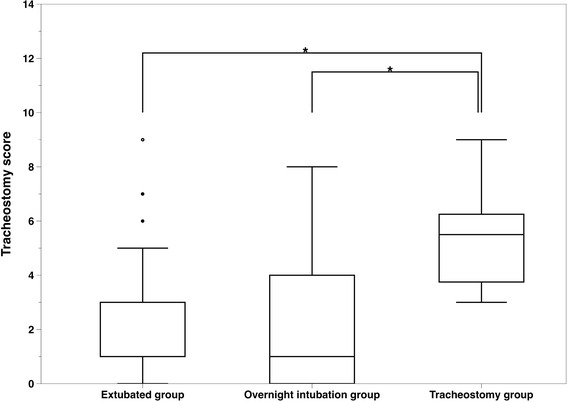
Variations in the tracheostomy scores between the airway management groups. (Data: box limits = upper/lower quartiles, error bars = max/min, line = median, outliers = •), *p < 0.05 (The Kruskal-Wallis test with multiple-comparison post test)

**Fig. 3:**
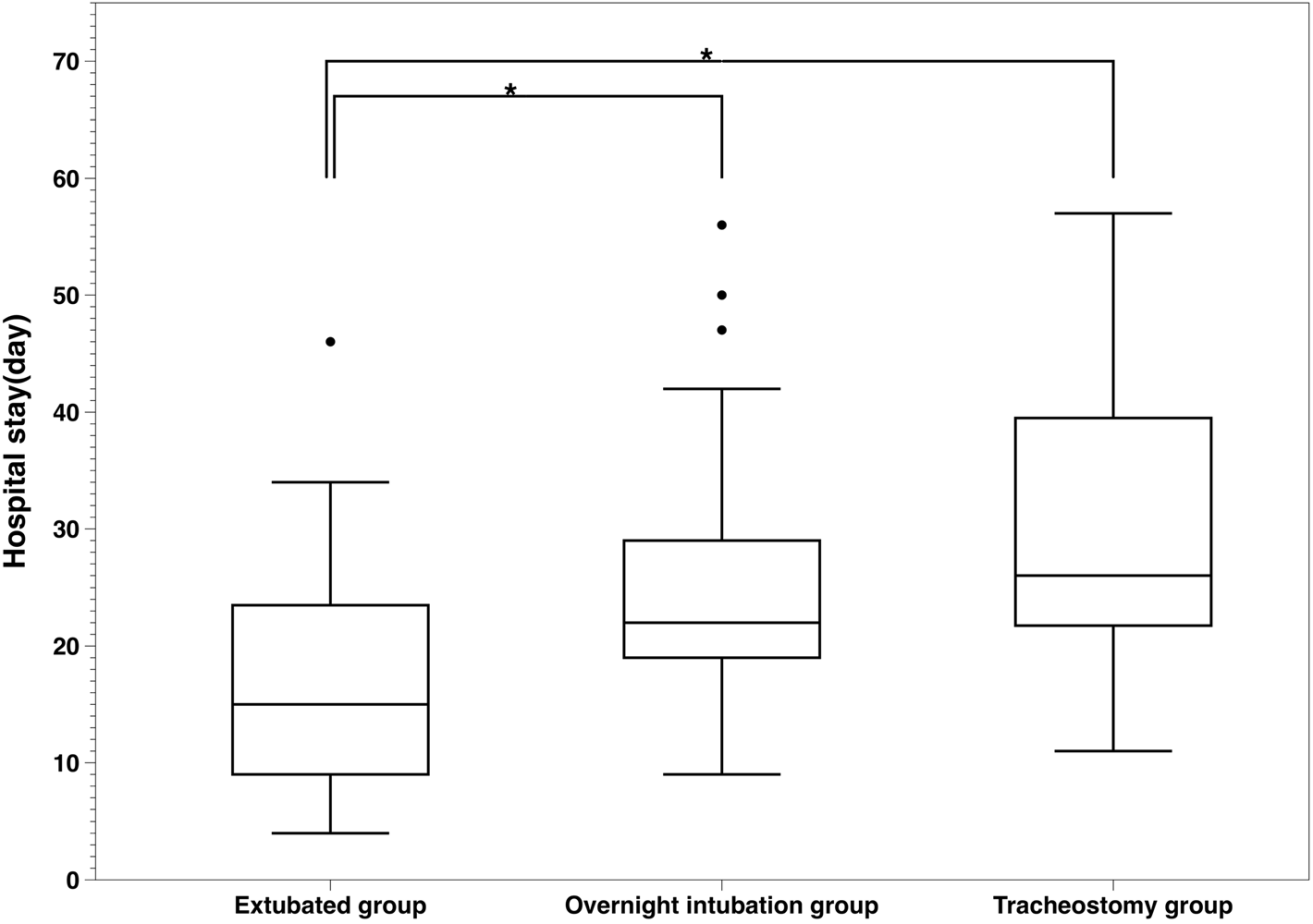
Variations in the length of hospital stay (days) between the airway management groups. (Data: box limits = upper/lower quartiles, error bars = max/min, line = median, outliers = •), *p < 0.05 (The Kruskal-Wallis test with multiple-comparison post test)

In the extubated group, only 6 patients out of 51 patients (11.8 %) had a score of more than 5 points. In the overnight intubation group, 11 patients out of 45 patients (24.4 %) had more than 5 points. There were significant differences in the number of patients for the three groups between the group with more than 5 points and the group with less than 5 points (*p* < 0.05). A total of 22 patients (21.2 %) had more than 5 points. But 17 patients of which did not have a tracheostomy and any postoperative emergency airway problems (Fig. 1). The tracheostomy score and length of hospital stay in each group showed significant differences (*p* < 0.05). For the tracheostomy score, there were significant differences between the extubated group and tracheostomy group and between the overnight intubation group and tracheostomy group (Fig. 2). For the length of the hospital stay, there were significant differences between the extubated group and overnight intubation group and between the extubated group and tracheostomy group (Fig. 3).

**Fig. 4:**
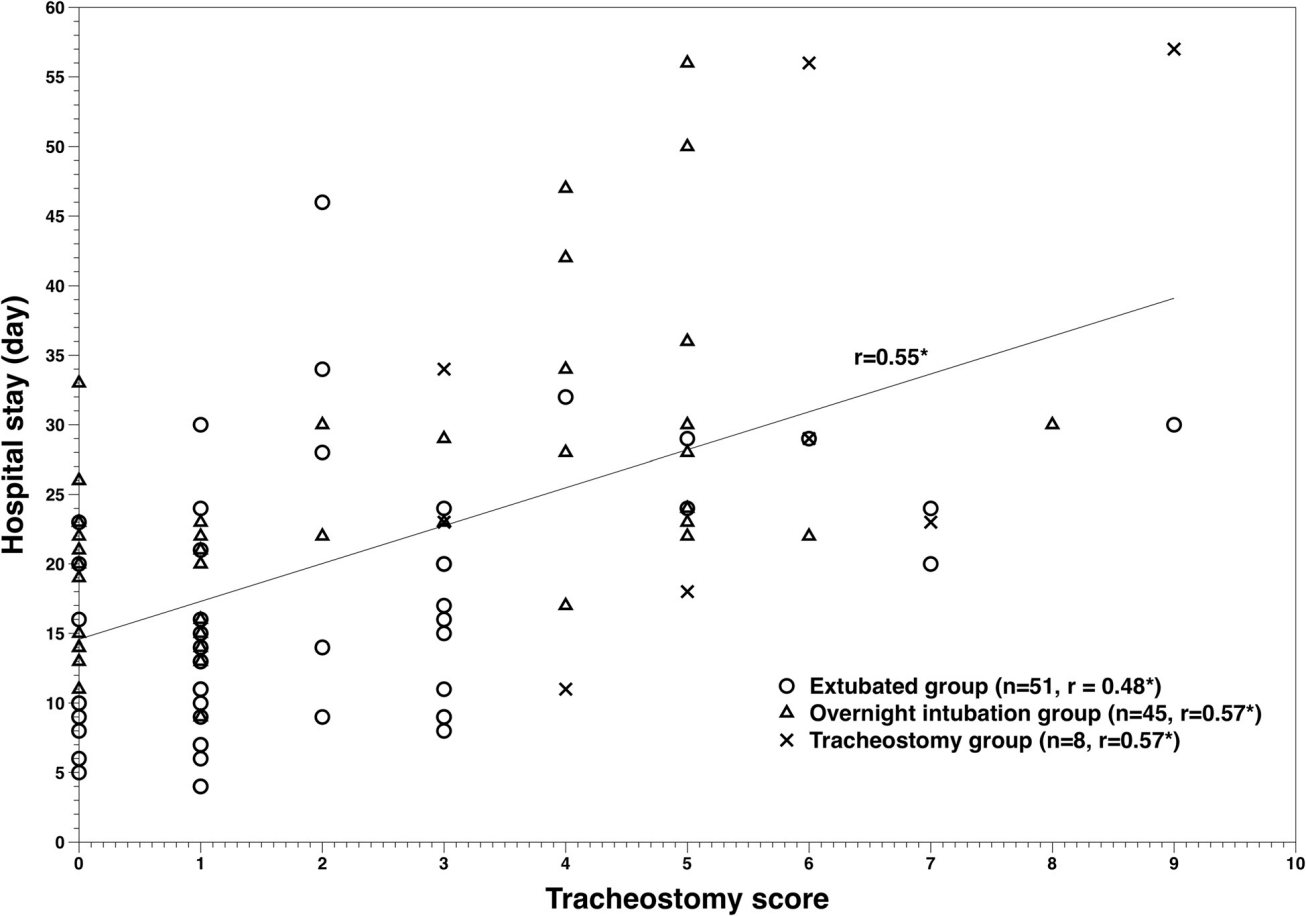
Correlation between the tracheostomy score and length of hospital stay. * : *P* < 0.05

A significant correlation (Pearson’s correlation coefficient r = 0.55; *p* < 0.05) was found between the tracheostomy score and length of hospital stay in all three groups (Fig. 4). The length of hospital stay between the group with more than 5 points and the group with less than 5 points was also significantly different (*p* < 0.05, Mann–Whitney *U*-test).

## Discussion

A tracheostomy is the most secure method to prevent an airway obstruction after the surgical treatment of head and neck cancer. A national survey in UK showed 69 % of clinical units (39/57) electively performed a tracheostomy ‘usually’ or ‘almost always’ after free flap head and neck reconstructive surgery [7]. A postoperative compromised airway is very difficult to manage. If there are some emergency situations, emergency intubation is difficult due to edema and bleeding in the oral cavity and neck. Usually in that situation, the patient is not under sedation or there is not enough time for sedation or to bring the patient to an operation room. Even a tracheostomy is difficult in these emergency situations. It is generally known that complications are more frequent in tracheostomies performed under emergency conditions [8]. If a patient has a possibility of compromised airway postoperatively, elective tracheostomy can be considered as a secure choice of treatment.

However, tracheostomy is also a traumatic procedure to the patients, which needs careful postoperative management. Tracheostomy-related complications are common and sometimes are life-threatening. Complications occurring from tracheostomy can be from 4.1 to 45 % [9–11]. Chest infections are common and those patients have a longer hospital stay [12]. The total hospital stay can be longer in patients because of the tracheostomy itself. Castling showed that patients with a tracheostomy-related complication had a mean total hospital stay of 25 days compared with 14 days for all patients [3]. Most tracheostomy related complications occur on the ward rather than in the ICU [13]. An increased length of hospital stay after tracheostomy is another factor to consider. The cost of the intensive care unit and the hospital stay can increase because of a tracheostomy. If complications occur, the cost will increase even more. In this study, the length of the hospital stay showed a significant positive correlation with the tracheostomy score. The extubated group had significantly shorter hospital stays compared with the other groups for hospital stay in this study. The tracheostomy scores include the aggressiveness of the operation such as reconstruction surgery and bilateral neck dissection as factors. The more aggressive the surgery was, the higher the tracheostomy scores were. The extubated group underwent relatively less aggressive surgery and had shorter hospital stays. The tracheostomy score can be used as a grading system for the severity of the oral cancer surgery for a clinical study.

Maintaining the intubation overnight after surgery can be one of the safe alternatives to a tracheostomy in oral cancer patients [14]. It can reduce the potential risk associated with a tracheostomy and result in a shorter recovery. However, the use of overnight intubation also has risks and needs careful postoperative management. The nurses (ICU or wards) should be experienced in the care of oral and maxillofacial surgery patients [15] because the nasotracheal tube can become obstructed easily from bleeding and mucous secretion and sometimes the patients have maxillomandibular fixation.

The period for maintaining endotracheal intubation is usually short (one or two days). If a longer period of intubation is expected, then a tracheostomy is recommended. Coyle reported that their 55 oral cancer patients were returned to the ICU being intubated without a tracheostomy and the intubation was maintained for the first postoperative night. Twenty-four patients (44 %) of the 55 patients had a score of 5 or more, which was considered to be the score at which an elective tracheostomy should be considered for the management of the airway. In this study, 8 patients (7.7 %) had a tracheostomy, and 22 patients (22/104, 21.2 %) had scores of more than 5 points. However many patients with high scores (17/22, 77.3 %) did not receive a tracheostomy. Five patients (5/8, 62.5 %) in the tracheostomy group had more than 5 points. The patient No. 6 had emergency problems during postoperative care. Other two patients (patients 7,8) had less than 5 points, but the operator considered the operation time and intraoperative bleeding and decided elective tracheostomy based on the clinical experience of the operator.

Cameron’s scoring system classified the factors that influence the decision for performing a tracheostomy in 4 key domains: tumor site, mandibulectomy, neck dissection, and reconstruction^6)^. They used a threshold score of 5 from the data of 143 patients (grouped into extubated at the end of the operation, overnight ventilation via an endotracheal tube, and elective tracheostomy) using Receiver operation characteristic (ROC) curve analysis. However, the results of this report showed that the scores of the patients in our clinic were not much in agreement with their report. Only 5 patients (22.7 %) among the 22 patients with more than 5 points for a Cameron score had a tracheostomy. The airway management was possible by maintaining overnight the intubation in the other patients. The tracheostomy score for the patient who had the nasotracheal tube on the second postoperative day and underwent an emergency tracheostomy was 3. From the result of this study, if we perform the elective tracheostomy with Cameron’s scoring system, there is possibility that more patients need tracheostomy unnecessarily. The differences of the results in this study can be explained with the limitation of the scoring system. Tumor size and location are important factors. Tumor size was not considered as a main factor in the Cameron’s scoring system. Tumor location such as anterior or posterior, buccal or lingual is also considered important in postoperative airway obstruction. Usually posterior and lingual side cancers have more complicated postoperative airway management. However, the airways of patients with cancer on the anterior mandible can be compromised despite its anterior location. Detachment of the genioglossus muscle, geniohyoid muscle, and mylohyoid muscle can be an aggravating factor in anterior midline cases. Bilateral neck dissection is also considered as one of the main factors for making an operator consider elective tracheostomy.

Another scoring system was introduced by Kruse-Losler [16]. They used the following 5 parameters: tumor localization (anterior and posterior to second premolars), tumor size (T1-4), Chest X-ray (with or without pathologic findings), multi morbidity (No or Yes), and alcohol consumption (No, <100g/day, >100g/day, hard drinks). An elective tracheostomy was recommended to a patient with more than 7 points. Their report showed that general medical condition and the level of alcohol consumption influenced significantly the decision for or against an elective tracheostomy. In this study, the Kruse-Losler’s scoring system could not be applied because the alcohol consumption data were not based on their criteria.

Predicting the postoperative airway state is difficult but it is one of the most important decisions for a safe and early recovery after oral cancer surgery. The airway was managed by maintaining the endotracheal intubation for 1 or 2 postoperative days in most cases in this study. Both Cameron’s score and Kruse’s score cannot be absolute guidelines in all cases. Using the scoring system was not sufficient to make a decision on whether to perform an elective tracheostomy after oral cancer surgery, but it can be helpful in predicting the severity of the airway obstruction after surgery.

The limitation of this study is that the decision whether to perform an elective tracheostomy or maintain the intubation in the patients of this study was not based on the scoring system, and only a retrospective study was done to review airway management according to the tracheostomy scoring system. Prospective studies are necessary to evaluate the predictive value of the scoring system.

## Conclusions

Predicting the necessity of elective tracheostomy is a difficult problem in a specific individual patient. In this study, the tracheostomy group showed a higher score according to Cameron’s grading system. But the airways of the patients could be managed postoperatively without a tracheostomy in most of the oral cancer patients even when their tracheostomy score was high. When postoperative airway management is anticipated to be difficult, an elective tracheostomy is the safest method. However, considering the complications and longer hospital stay from a tracheostomy, maintaining the nasotracheal intubation overnight is a good alternative to a tracheostomy. The indication of postoperative elective tracheostomy may be different in each operator. The scoring system did not quite agree with the airway management of the authors’ clinic; however, it can be one of the clinical factors predicting the degree of the postoperative airway obstruction and surgical aggressiveness for recovery. The further studies are needed for clinically more reliable scoring systems.

## Consent

Written informed consent was obtained from the patient for the publication of this report and any accompanying images.
